# Significance of Competing Metabolic Pathways for 5F-APINACA Based on Quantitative Kinetics

**DOI:** 10.3390/molecules25204820

**Published:** 2020-10-20

**Authors:** Anna O. Pinson, Dakota L. Pouncey, Mary A. Schleiff, William E. Fantegrossi, Paul L. Prather, Anna Radominska-Pandya, Gunnar Boysen, Grover P. Miller

**Affiliations:** 1 Department of Chemistry and Biochemistry, Harding University, Searcy, AR 72149, USA; apinson@harding.edu; 2 College of Medicine, University of Arkansas for Medical Sciences, Little Rock, AR 72205, USA; DLPouncey@uams.edu; 3 Department of Biochemistry and Molecular Biology, University of Arkansas for Medical Sciences, Little Rock, AR 72205, USA; MADavis@uams.edu (M.A.S.); RadominskaAnna@uams.edu (A.R.-P.); 4 Department of Pharmacology and Toxicology, University of Arkansas for Medical Sciences, Little Rock, AR 72205, USA; WEFantegrossi@uams.edu (W.E.F.); PratherPaulL@uams.edu (P.L.P.); 5 Department of Environmental and Occupational Health, University of Arkansas for Medical Sciences, Little Rock, AR 72205, USA; GBoysen@uams.edu

**Keywords:** synthetic cannabinoid, 5F-APINACA, 5F-AKB48, P450, kinetics, drug abuse, CB1 receptor

## Abstract

In 2020, nearly one-third of new drugs on the global market were synthetic cannabinoids including the drug of abuse *N*-(1-adamantyl)-1-(5-pentyl)-1*H*-indazole-3-carboxamide (5F-APINACA, 5F-AKB48). Knowledge of 5F-APINACA metabolism provides a critical mechanistic basis to interpret and predict abuser outcomes. Prior qualitative studies identified which metabolic processes occur but not the order and extent of them and often relied on problematic “semi-quantitative” mass spectroscopic (MS) approaches. We capitalized on 5F-APINACA absorbance for quantitation while leveraging MS to characterize metabolite structures for measuring 5F-APINACA steady-state kinetics. We demonstrated the reliability of absorbance and not MS for inferring metabolite levels. Human liver microsomal reactions yielded eight metabolites by MS but only five by absorbance. Subsequent kinetic studies on primary and secondary metabolites revealed highly efficient mono- and dihydroxylation of the adamantyl group and much less efficient oxidative defluorination at the *N*-pentyl terminus. Based on regiospecificity and kinetics, we constructed pathways for competing and intersecting steps in 5F-APINACA metabolism. Overall efficiency for adamantyl oxidation was 17-fold higher than that for oxidative defluorination, showing significant bias in metabolic flux and subsequent metabolite profile compositions. Lastly, our analytical approach provides a powerful new strategy to more accurately assess metabolic kinetics for other understudied synthetic cannabinoids possessing the indazole chromophore.

## 1. Introduction

Synthetic cannabinoid abuse has become the center of a drug crisis in the past decade. As of January 2020, synthetic cannabinoids account for nearly one-third of all new drugs appearing on the global market [1]. In the United States, it was recently estimated that 36 million people have experimented with these drugs in their lives [2]. A driver in this crisis is the constant evolution of newer generations of illicit drugs to evade detection and litigation, thus leading to creation of the *N*-(1-alkyl)-1-pentyl-1*H*-indazole-3-carboxamide family of synthetic cannabinoids. These potent drugs share a central indazole core modified with an *N*-pentyl group and linked to a second alkyl group through a carbonyl bond. A later generation of the drug involved introduction of fluorine at the pentyl terminus, creating *N*-(1-adamantyl)-1-(5-pentyl)-1*H*-indazole-3-carboxamide (5F-APINACA, also known as 5F-AKB48). The fluorine increases drug lipophilicity to increase drug distribution across the blood–brain barrier, and thus improve access and high-affinity [3,4,5] binding to cannabinoid type 1 receptors (CB1Rs) in the central nervous system, as shown by *in vitro* cell culture [3,6] and *in vivo* rodent studies [7,8]. Those qualities may facilitate the toxic effects such as nausea, vomiting, tachycardia, and occasionally death resulting from exposure [9]. On the other hand, metabolism counters these effects by altering drug structure, often resulting in decreased affinity toward CB1Rs [6] and facilitating elimination. Knowledge of which metabolic pathways exist and their relative significance is then critical for understanding biological and toxicological responses to 5F-APINACA and other synthetic cannabinoid exposures. Moreover, metabolic information will likely provide a critical mechanistic basis for interpreting and predicting the impact of clinical factors such as age, sex, genetic polymorphisms, and drug–drug interactions on 5F-APINACA response among abusers.

5F-APINACA undergoes extensive metabolism including oxidative defluorination of the *N*-pentyl group and hydroxylation of the adamantyl moiety. In vitro studies with human liver microsomes provide insights into the occurrence of those reactions and enzymes likely responsible for them [8,10,11]. Presence of the resulting metabolites in urine surveys from abusers provides clinical evidence for those specific metabolic pathways [10,12]. Those analyses did not involve authentic standards for 5F-APINACA metabolites due to their lack of availability. Generation of standards incurs significant investments in time, effort and resources, and thus this approach is not practical for widespread use in studies. Consequently, 5F-APINACA studies rely on inference of metabolite levels assuming the mass spectroscopic (MS) response is the same among them and the parent drug. Nevertheless, other research studies with a structurally diverse array of drugs and associated metabolites have shown that this assumption is not true in most cases [13,14]. Specifically, MS response varies significantly among metabolites and parent drugs, so “semi-quantitative” studies using MS response to infer levels of metabolites relative to one another often lead to inaccurate results. In following, prior research on 5F-APINACA metabolism identified possible metabolic pathways but not the relative significance of those steps. Kinetic studies provide a tractable approach to scale the efficiencies of reactions impacting drug structure and hence, potency and response; however, published microsomal studies for 5F-APINACA were not carried out under steady-state conditions [8,10,11], so the relative efficiencies of reaction steps and pathways, and thus their relevance remain unknown.

As an alternative for metabolic studies on synthetic cannabinoids, we capitalized on the spectral properties of 5F-APINACA for quantitation while leveraging MS techniques to characterize individual metabolite structures. Like many synthetic cannabinoids, 5F-APINACA possesses a central indazole core with absorbance and fluorescence properties suitable for quantitation. If metabolism does not alter the chromophore, then the spectral response does not change for the metabolites relative to substrate. In following, substrate spectroscopic response can be used for inferring the quantitation of its metabolites in the absence of authentic standards for them. Reports validate the rigor of this approach in multiple metabolism studies [15,16,17] including applications in drug metabolism, such as glucuronidation by UDP-glucuronosyltransferases [18]. In practice, we employ a two-step strategy for estimating metabolite levels from microsomal reactions. First, we confirm the absence of chromophore modifications using MS techniques to characterize metabolite structures. Second, we chromatographically resolve metabolites to visualize their respective responses based on absorbance and fluorescence. We can then infer metabolite quantitation using the corresponding spectral response of the substrate. For this study, we hypothesized that this approach would yield steady-state mechanisms and constants describing efficiencies of metabolic steps that collectively determine the importance of competing and intersecting metabolic pathways for 5F-APINACA.

Herein, we are the first to measure steady-state kinetics for 5F-APINACA using a powerful combination of analytical approaches that obviated the need for authentic metabolite standards. As a model for the average adult liver, we carried out metabolic reactions with 5F-APINACA using pooled human liver microsomes. After establishing steady-state conditions, a critical early step involved maximizing chromatographic resolution of individual metabolites to make quantitation by absorbance and fluorescence possible. Next, we characterized metabolite structures by MS and ascribed them to steps within metabolic pathways. In the process, we determined whether these reactions altered the indazole chromophore as an assessment of the reliability of inferring metabolite levels based on the absorbance or fluorescence response of the substrate. Subsequent steady-state analyses for 5F-APINACA provided insights on the mechanisms and constants determining the relative significance of steps in its overall metabolism. Collectively, we were then able to use the kinetic data for constructing the network of competing and intersecting metabolic steps that determine the metabolic flux of 5F-APINACA down detoxification and clearance pathways.

## 2. Results

### 2.1. Absorbance but Not MS Proved Reliable for Inferring Quantitation of Five 5F-APINACA Metabolites

**Figure 1:**
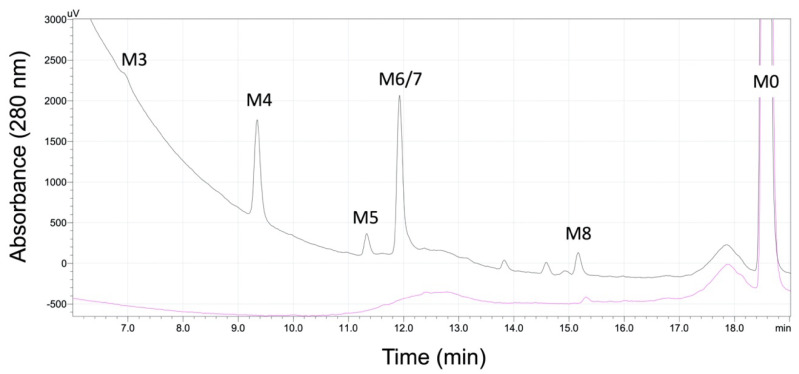
Chromatographic resolution of 5F-APINACA and metabolites as detected by absorbance. We resolved and analyzed analytes from a 250 μM 5F-APINACA reaction to identify potential metabolites using a Shimadzu UHFLC was equipped with an SPD-10A UV–Vis (280 nm) and RF-10AXL fluorescence (excitation 280 nm, emission 650 nm) detectors. The chromatogram for the reaction in the absence of the NADPH-regenerating system is shown in pink and the chromatogram from the reaction with the system shown in black. The resulting chromatogram shows absorbance peaks for five metabolites (M3, M4, M5, M6/M7, and M8) and parent 5F-APINACA (M0). M6 and M7 co-eluted when monitoring absorbance but were resolved when detecting by MS (see Results for details). Of the metabolites, M8 formed during reactions with and without NADPH and so was present in both chromatograms. Analyte numbering reflects more extensive detection of possible metabolites using the Agilent Technologic 1290 Infinity HPLC equipped with a 6490 Triple Quad MS (see Figure 2 and Figure A3).

**Figure A1:**
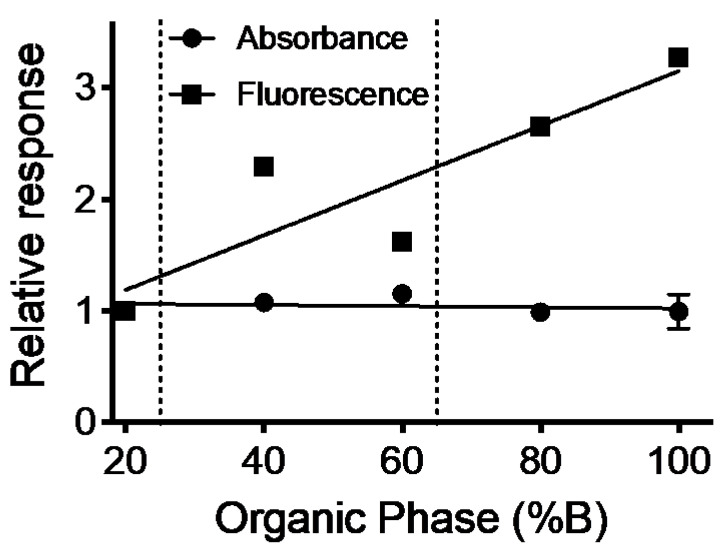
5F-APINACA absorbance and fluorescence response as function of organic-phase content. Analytes from 5F-APINACA metabolism eluted during a gradient between solvent A (10% solvent B, 90% water) and solvent B (0.1% formic acid/acetonitrile) in which solvent B increased from 25% to 65%. A high organic phase could impact spectroscopic response, so we prepared a 5 μM 5F-APINACA solution with increasing solvent B and measured its absorbance (280 nm) and fluorescence (excitation 280 nm; emission 650 nm). Data were normalized to response at 20% solvent B as baseline, plotted and then fit to a linear regression using GraphPad Prism 8.0 from GraphPad Software, Inc (San Diego, CA, USA). The dashed lines indicate the range of the organic phase during the chromatographic gradient in which the analytes elute during the run.

We carried out and analyzed initial metabolic reactions for 5F-APINACA to develop a chromatographic method for maximizing analyte resolution, and thus render identification and quantitative assessment of metabolites possible. The use of a spectroscopic signal for inferring quantitation requires the spectral response to be independent of analytical conditions for all analytes. As a test, we determined the effect of organic solvent (1:1 methanol:acetonitrile) on absorbance and fluorescence of 5F-APINACA as expected during chromatographic resolution of analytes. The fluorescence response increased positively and linearly with the percent of organic solvents while absorbance response did not change significantly (Figure A1). Consequently, all quantitative inference for kinetic studies relied on the absorbance response, which was similar in sensitivity to that for fluorescence. After optimization analyte resolution, the final chromatogram in Figure 1 shows absorbance peaks for 5F-APINACA (M0) and five metabolites.

**Figure A2:**
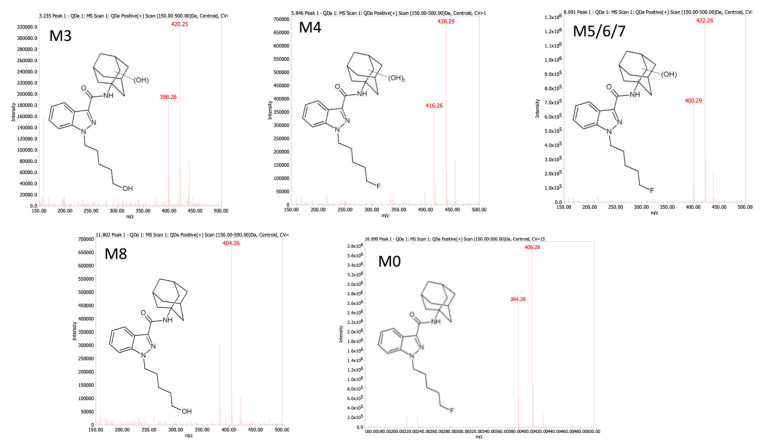
MS spectra for analyte parent masses corresponding to fluorescence peaks from 5F-APINACA microsomal reaction. We resolved and analyzed metabolites from a 500 μM 5F-APINACA reaction to correlate fluorescent peaks to parent masses for analytes using a Waters Acquity UPLC-Fluorescence/QDa (single quadrupole) instrument. Major ions reflect parent masses and sodium adducts (*m*/*z*). Both [MH]^+^ and [M + Na]^+^ values are indicated in the figures.

From the QDa single quadrupole detector, the corresponding parent masses (*m*/*z*) for five analytes are listed in Table 1 and spectra shown in Figure A2. Differences in mass between metabolites and parent drug were consistent with oxidative defluorination and hydroxylations; however, it was not possible to assign regiospecificity of hydroxylations based solely on parent mass. We then ported this chromatographic method to our Agilent Technologic 1290 Infinity HPLC with a 6490 Triple Quad MS, which has higher sensitivity and the capacity to fragment ions for a more thorough analysis. There was a slight shift in retention time for analytes between the instruments, yet the pattern and parent masses matched those from the previously determined chromatogram. This secondary analysis yielded eight observable metabolites and the parent drug serving as the basis for numbering metabolites in this study. By comparison, the lower number of observable metabolites by absorbance and fluorescence indicated that (1) M1 and M2 were low-yield metabolites below the detection limit of the detectors and (2) M6 and M7 co-eluted into a single peak (hence, the label M6/M7 in further discussions).

We characterized specific metabolites from 5F-APINACA reactions based on reported characteristic MS features [8,10,19,20]. First, loss of the adamantyl group (*m*/*z* 153.1) is a dominant fragment ion for unmetabolized 5F-APINACA as well as its metabolites. Monohydroxylation of the adamantyl group yields fragment ions at *m*/*z* 151.1 for the monohydroxy adamantyl cation and *m*/*z* 133.1 after the sequential loss of a water molecule. The pattern for the dihydroxy adamantyl group is *m*/*z* 167.1 with *m*/*z* 149.1 and *m*/*z* 131.1 ions reflecting sequential losses of water. Second, oxidative defluorination of 5F-APINACA yielded the loss of fluorine and addition of a hydroxyl group at the terminus of the N-pentyl group. Unfortunately, our attempts to obtain fragments other than the N-fluoropentyl indazole acylium ion (*m*/*z* 233.0) were unsuccessful in line with observations by others [8,10,19,20].

**Figure 2:**
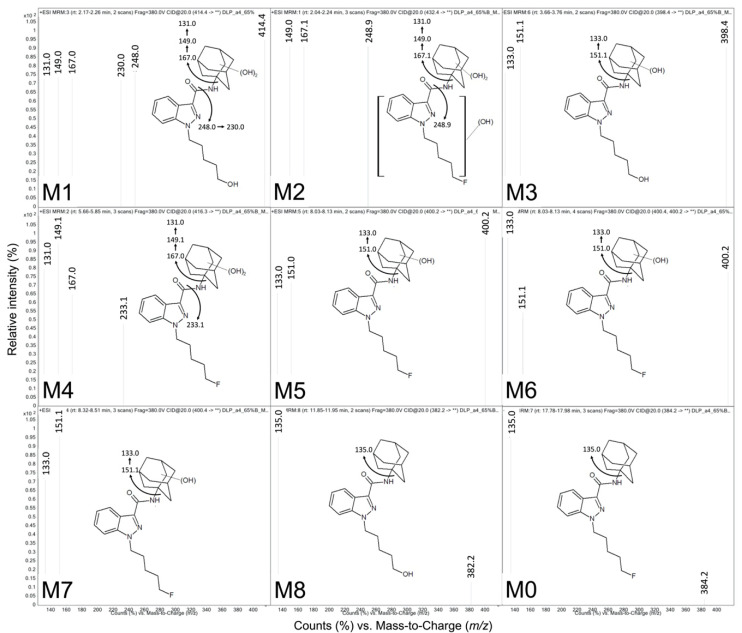
MS fragmentation spectra for 5F-APINACA metabolites and proposed structures. We resolved and analyzed metabolites from a 200 μM 5F-APINACA reaction to characterize their structures using an Agilent Technologic 1290 Infinity HPLC equipped with a 6490 Triple Quad MS. The resulting product ion chromatograms are shown in Figure A3 and the above figure indicates the corresponding fragmentation patterns of each analyte. Signature ions are summarized in Table 1 and were used in the assignment of putative structures as described previously [8,10,11,20].

**Figure A3:**
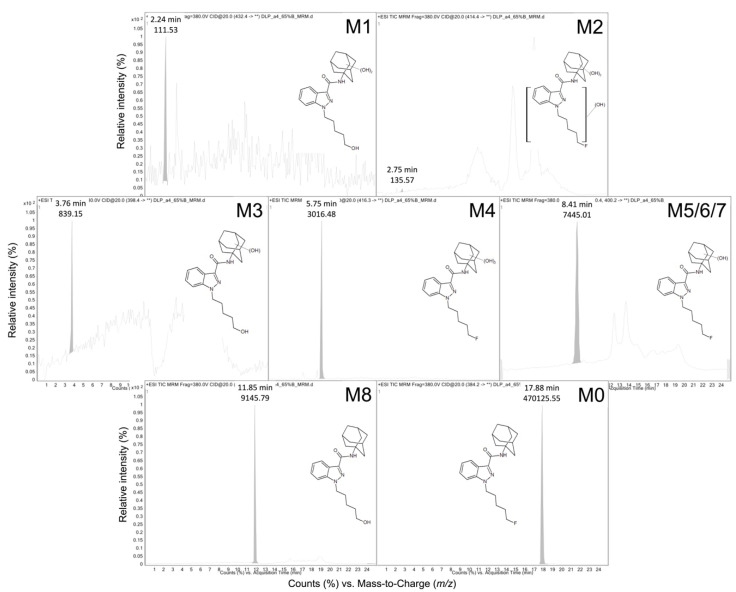
Extracted ion chromatograms from the total ion scan for all analytes. We resolved and analyzed metabolites from a 200 μM 5F-APINACA reaction to characterize their structures using an Agilent Technologic 1290 Infinity HPLC equipped with a 6490 Triple Quad MS. The above figure shows the extracted ion chromatograms from the total ion scan for all analytes. Subsequent MRM analysis of each analyte yielded fragmentation patterns indicated in Figure 2 and summarized in Table 1. M5 and M7 were visualized by MRM analysis but could not be visualized in the TIC due to poor signals. Signature ions are summarized in Table 1 and were used in the assignment of putative structures as described by previously [8,10,20].

The observed fragment ions and details are listed in Figure 2 and Table 1, while parent mass peaks from the product ion chromatograms are included in Figure A3. M3, M5, M6 and M7 metabolites possessed fragment ions indicating monohydroxylation of the adamantyl group, while M1, M2, and M4 metabolites shared the pattern for dihydroxylation of the adamantyl moiety. The differences in parent mass from the parent drug to M1, M3 and M8 were consistent with oxidative defluorination. M1 displayed a probable dehydration reaction of the 248.0 *m*/*z* fragment ion to yield one at 230.0 *m*/*z*. For M2, there was an additional hydroxylation distal to the adamantyl ring, and thus residing on the indazole or *N*-alkyl group. The parent drug possessed the expected fragment ion for the unmodified adamantyl group. Moreover, the same fragmentation pattern and similar elution times for M5, M6 and M7 suggests that they are hydroxy adamantyl isomers as observed in a previous study [10]. The identity and order of elution of these metabolites matched previous reports under similar chromatographic conditions [8,10,11] as further support for their assignments. Taken together, we classified the order of metabolites as primary (M5, M6, M7 and M8), secondary (M3 and M4), and tertiary (M1 and M2) based on the respective structural modifications. Importantly, all metabolite peaks observed by absorbance and fluorescence corresponded to metabolites lacking modification of the indazole ring, and thus we can accurately infer quantitation of metabolites for kinetic studies based on the indazole chromophore.

**Figure 3:**
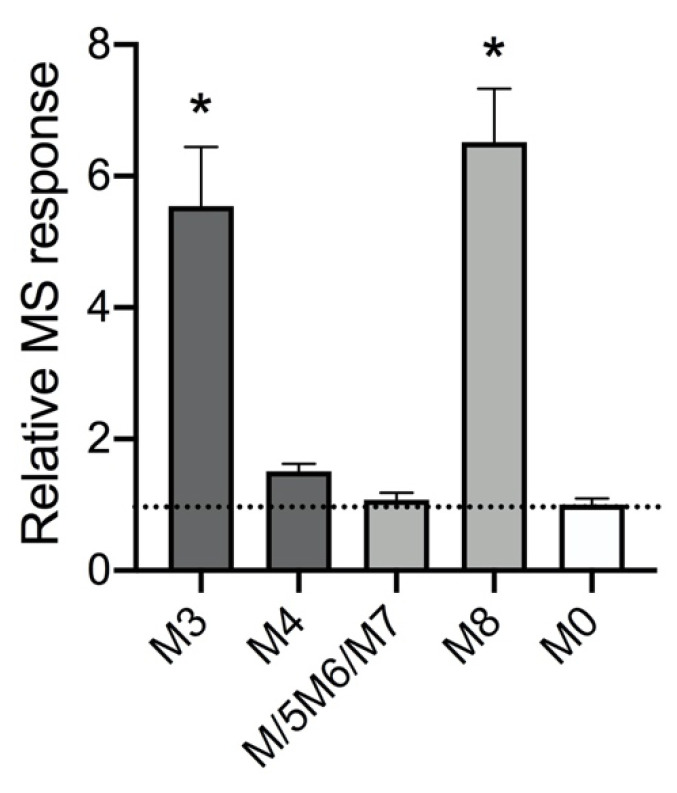
Relative MS responses for 5F-APINACA metabolites. We analyzed metabolites from a 200 μM 5F-APINACA reaction as described under Methods and then used values to assess relative MS responses for metabolites. Calculation of the metabolite MS responses involved dividing respective peak areas from the extracted ion chromatograms generated from the total ion scan (Figure A3) by the metabolite concentration as inferred from absorbance. The resulting values for metabolites were then normalized to parent drug response as a measure of their relative variability. This analysis was possible only for metabolites observable by absorbance, i.e., parent (M0) in white, primary metabolites (M5/M6/M7 and M8) in light gray, and secondary metabolites (M3 and M4) in dark gray. For each metabolite, values reflect data from six replicates. Statistical differences among final responses were then calculated using ordinary one-way ANOVA analysis comparing metabolite responses to parent (M0) yielding a *p* value of <0.0001 and indicated by asterisks (*).

Given this knowledge, we assessed MS responses among the metabolites relative to the parent drug as employed in common “semi-quantitative” metabolism studies [13,14]. After conducting a 200 μM 5F-APINACA reaction, peak areas for each analyte were calculated from the corresponding extracted ion chromatogram generated with the total ion scan (Figure A3) and then divided by concentrations as inferred from analyte analyses by absorbance (vide supra). Resulting MS response values were then normalized to parent drug response as a measure of their relative variability. This analysis was possible only for metabolites observable by absorbance, i.e., parent (M0), primary metabolites (M5/M6/M7 and M8), and secondary metabolites (M3 and M4). Peak areas were summed together for all three hydroxy adamantyl metabolites (M5/M6/M7) due to co-elution of M6 and M7 when measuring absorbance and observation of only M6 by MS. As shown in Figure 3, the type and/or location of metabolism impacted the MS response for metabolites when compared to the parent drug. Oxidative defluorination (M3 and M8) led to an approximate 6-fold increase in response relative to parent, while hydroxylation of the adamantyl group had little to no impact on MS response (M4 and M5/M6/M7) (*p* < 0.0001 based on one-way ANOVA). Taken together, significant variability in metabolite MS responses relative to parent 5F-APINACA demonstrated the unreliability of MS response for inferring metabolite levels.

### 2.2. Steady-State Kinetics Revealed Relative Importance of Binding and Chemistry in Metabolism

**Figure 4:**
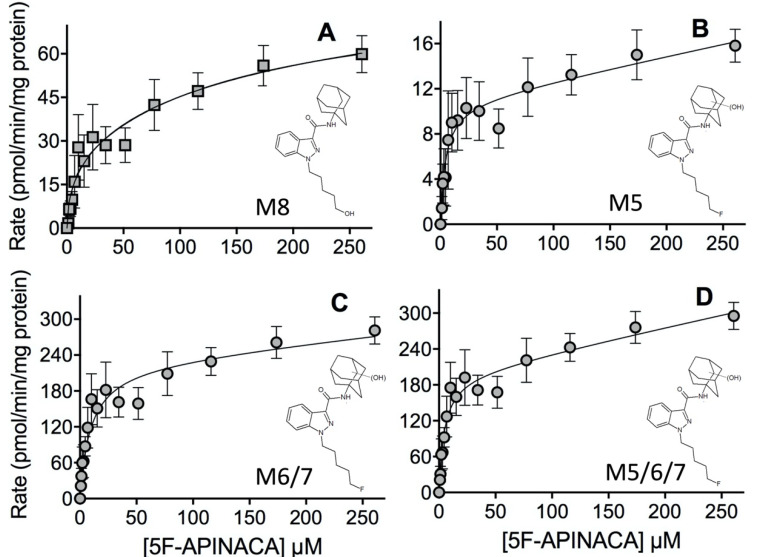
Steady-state kinetic profiles for 5F-APINACA primary metabolites from microsomal reactions. (**A**) Panel shows the kinetic data for oxidative defluorination of 5F-APINACA into M8 (squares, light gray), while panels in (**B**,**C**) reflect results for the early and late eluting monohydroxy adamantyl metabolite isomers M5 and M6/M7 (circles, light gray). (**D**) Both data sets were combined to generate the kinetic profile and analyzed as a reflection of total monohydroxylation of adamantyl group of 5F-APINACA (circles, light gray). Steady-state reaction conditions and data analyses were carried out as described in Materials and Methods. Each data point is the average of seven replicates from three separate experiments and the displayed curve reflects the best-fit model for the data. The corresponding mechanism and constants are reported in Table 2.

Under steady-state conditions, we measured kinetics for primary and secondary metabolites of 5F-APINACA metabolism. Trace levels of tertiary metabolites were detectable, but their responses were not sufficient for measuring kinetics. Initial control experiments revealed that oxidative defluorination occurred in the absence of NADPH, which is typically used to initiate reactions. Thus, substrate was added last for more accurate measures of initial reaction rates. Oxidative defluorination (M8) was biphasic reflecting the contribution of two kinetically distinct activities (Figure 4, Panel A; Table 2). The maximal turnover rate (V_max_) for the higher affinity activity was one-third of that for the lower affinity activity. Despite a higher V_max_, the K_m_ was 35-fold higher than the higher affinity activity, making it much less efficient.

Monohydroxylation of the adamantyl group led to formation of isomers (M5 and M6/M7) with the same kinetic mechanisms but with different kinetic constants. The kinetic profiles reflected a high-affinity, saturable metabolism that fit to a Michaelis–Menten model followed by an unsaturable, low capacity linear contribution (Figure 4, Panels B and C; Table 2). The earlier eluting M5 bound to enzyme with high affinity that led to low maximal turnover. In contrast, the metabolite isomers (M6/M7) were metabolized at a 20-fold higher rate, with only a 2-fold decrease in binding (K_m_), so this pathway was much more efficient. Of note, the secondary MS analysis resolved all three isomers and indicated that the response for M6 was much higher than that for M5 and M7 indicating a clear preference in regiospecificity for the reaction. Taken together, these primary metabolites reflect a common first step in 5F-APINACA metabolism, and thus we combined data sets and determined the mechanism and constants as a global assessment of this reaction step. The respective data sets differed in magnitude, so we added average rates at each substrate concentration and then propagated the corresponding standard deviations for the final plot (Figure 4, Panel D). These data fit best to the same mechanism as for individual metabolites reflecting a high-affinity and a saturable turnover rate followed by an unsaturable linear rate (Table 2).

**Figure 5:**
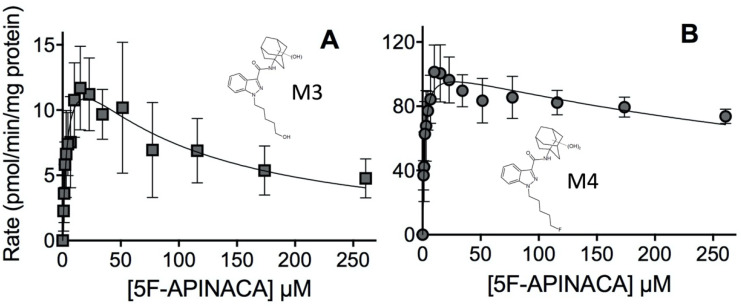
Steady-state kinetic profiles for 5F-APINACA secondary metabolites from microsomal reactions. (**A**) Panel reveals the kinetic data for M3 reflecting the combination of oxidative defluorination and adamantyl monohydroxylation of 5F-APINACA (squares, dark gray). (**B**) Panel shows the kinetic profile for the dihydroxy adamantyl metabolite (M4; circles, dark gray). Steady-state reaction conditions and data analyses were carried out as described in Materials and Methods. Each data point is the average of seven replicates from three separate experiments and the displayed curve reflects the best-fit model for the data. The corresponding mechanism and constants are reported in Table 2.

**Figure 6:**
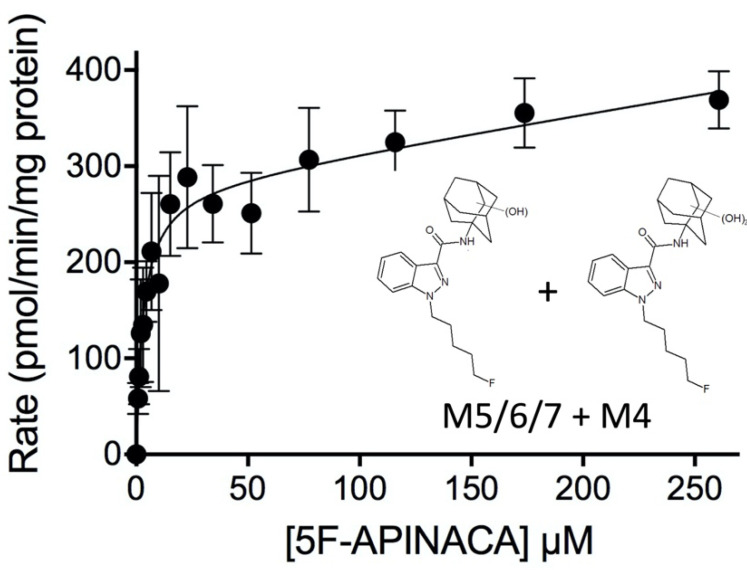
Kinetic profile for overall oxidation of the adamantyl group of 5F-APINACA. The kinetic data for mono- and dihydroxylation of 5F-APINACA (M4, M5, and M6/M7) were combined into one kinetic profile for analyzing the overall efficiency of adamantyl oxidation. The displayed curve reflects the best-fit model for the data. The corresponding mechanism and constants are reported in Table 2.

The kinetics for both secondary metabolites (M3 and M4) involved substrate inhibition. The combination of oxidative defluorination and adamantyl monohydroxylation involved high-affinity reactions but low turnover as well as moderate substrate inhibition (Figure 5, Panel A; Table 2). These kinetics for M3 reflected the least efficient step in 5F-APINACA metabolism under steady-state reaction conditions. On the other hand, further hydroxylation of M5 or M6/M7 led to the dihydroxy adamantyl metabolite (M4). The kinetics corresponded to the lowest K_m_ value in this study and a high maximal rate of turnover followed by a very weak contribution of substrate binding (Figure 5, Panel B; Table 2). Given this reaction requires the initial hydroxylation step, we combined and analyzed kinetic data for M4, M5 and M6/M7 to determine the efficiency of the overall pathway for adamantyl hydroxylation as shown in Figure 6 and Table 2. The efficiency of this reaction (V_max_/K_m_, 91) is 17-fold greater than that observed for oxidative defluorination (V_max_/K_m_, 5.2) among the competing 5F-APINACA metabolic pathways.

## 3. Discussion

### 3.1. New Quantitative Approach Yielded Steady-State Kinetics for 5F-APINACA Metabolism

Access to authentic standards for quantitative metabolism studies remains a challenge for the rapidly evolving field of illicit synthetic cannabinoids, and thus we explored the feasibility of a novel inference approach as a solution. Initially, we confirmed metabolic reactions did not alter the structure of the indazole chromophore. This outcome ensured that the indazole absorbance and fluorescence responses would remain equivalent among substrate and metabolites, thus making inference of metabolite levels from substrate response possible. Nevertheless, 5F-APINACA fluorescence was impacted by increasing concentration of organic solvents used in the chromatographic separation of the substrate and its metabolites. The use of fluorescence for inferring quantitation would then require a correction factor to adjust responses among the analytes and likely introduce potential error in the process. For simplicity, we relied solely on the absorbance response for inferring the quantitation of 5F-APINACA and its metabolites. Moreover, we showed high variability in relative MS response for these analytes presumably due to differences in ionization. This quality highlights how “semi-quantitative” mass spectral techniques are not conducive to inferring metabolite levels as demonstrated by others using more extensive arrays of drugs and metabolites [13,14]. The use of authentic standards then remains necessary for reliable quantitative studies by MS. Based on our findings, we demonstrated the reliability of absorbance for inferring 5F-APINACA levels from metabolic reactions and leveraged that capacity to generate the first reported steady-state kinetics for the drug in the absence of authentic metabolite standards.

### 3.2. Steady-State Kinetics Revealed Major and Minor Metabolic Pathways for 5F-APINACA

Based on reaction regiospecificity and kinetics, we constructed plausible pathways for competing and intersecting steps in 5F-APINACA metabolism. Unlike previously reported studies [8,10,11], our emphasis on quantitative kinetics provides an effective strategy to order metabolic steps and scale their relative significance in metabolic pathways. We estimated the flux through each metabolic step using catalytic efficiencies (V_max_/K_m_, pmol/min/mg protein/μM product) for intrinsic clearance (Cl_int_) based on calculations from data in Table 2. We biased selection for kinetic constants reflecting higher affinity activities due to typical exposures to nanomolar 5F-APINACA plasma levels in abusers [12]. Of note, those kinetic values reflect the dependency of rates on parent drug concentrations and not the relationship of specific substrates for reactions such as primary metabolite conversion into secondary metabolites. Consequently, kinetic mechanisms and constants are limited to 5F-APINACA levels under current reaction conditions providing suitable assessment of preferences among reaction steps and ultimately pathways relative to one another.

**Figure 7:**
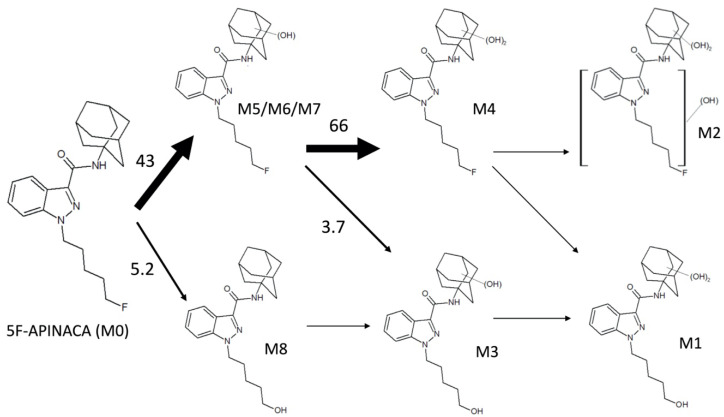
5F-APINACA metabolic flux through competing and intersecting pathways. We estimated 5F-APINACA metabolic flux through primary and secondary metabolic steps using catalytic efficiencies (V_max_/K_m_, pmol/min/mg protein/μM product) for intrinsic clearance (Cl_int_) with data in Table 2 and biasing selection for higher affinity activities due to typical exposures to low 5F-APINACA levels in abusers [12]. M3 originates from either M8 or M5/M6/M7; however, levels of the latter metabolites vastly exceeded those of M8, making the observed kinetics more likely due to conversion of M5/M6/M7 to M3 as shown. Downstream tertiary metabolites M1 and M2 were observed but lacked kinetics, and thus their relative significance was not measurable. Taken together, the magnitude of the values reflects the preference for 5F-APINACA to proceed through indicated metabolic steps.

Overall, there are two competing pathways in 5F-APINACA metabolism (Figure 7). Initially, the drug undergoes either oxidative defluorination (M8) or the significantly favored hydroxylation of the adamantyl group (M5/M6). The hydroxy adamantyl metabolite serves as an efficient substrate for a second hydroxylation of adamantyl group (M4). Based on these studies, it is unclear whether this reaction involves a release and rebinding event for the hydroxy adamantyl metabolite or the molecule remains bound to enzyme for a rapid sequential hydroxylation to the dihydroxy adamantyl metabolite. If released from the enzyme, then the hydroxy adamantyl metabolite could undergo oxidative defluorination to yield M3. Alternatively, that metabolite may arise from monohydroxylation of M8 after oxidative defluorination. This reaction seems more plausible given the significant efficiency of hydroxylation of the adamantyl group, and thus higher levels of the corresponding metabolite. The similarity in reaction kinetics for M3 and M8 is consistent with this possibility. We further expand the metabolic pathways to downstream tertiary metabolites M1 and M2, although kinetics were not measurable to assess their relative significance. For a more global perspective of 5F-APINACA metabolism, the overall efficiency for adamantyl oxidation is 17-fold higher than that for oxidative defluorination showing significant bias in the metabolic flux and subsequent composition of the metabolite profile.

### 3.3. 5F-APINACA Kinetics Supported Extrapolating the Potential Relevance of Metabolism on Abuser Responses

Metabolic pathways alter 5F-APINACA structure, thus contributing to clearance (pharmacokinetics) and potency (pharmacodynamics) that collectively determine drug response for abusers. Controlled pharmacokinetic studies are not possible for this illicit drug, making the extrapolation of findings from *in vitro* and *in vivo* model systems essential. Based on our microsomal kinetic studies, 5F-APINACA primarily undergoes efficient hydroxylation of the adamantyl group as the main determinant of clearance. The resulting metabolite accumulation then leads to subsequent, less efficient metabolism of the *N*-pentyl group. The eight primary, secondary and tertiary metabolites reported in our study were observed in surveys that relied on MS analyses of urine from abusers [10,11]. In those studies, precursor ion peak areas were relatively similar among metabolites with the exception of a significantly larger peak for defluorination and eventual carboxylation of the *N*-pentyl terminus. Without metabolite standards, their relative levels remain unknown; however, our current findings indicate lower MS sensitivity for hydroxy adamantyl metabolites and higher sensitivity toward metabolites with an oxidized *N*-pentyl group. Application of these trends to urine profiles for abusers suggests elevated levels of hydroxy adamantyl metabolites and much lower levels of metabolites modified at the *N*-pentyl terminus. This observation is consistent with our predictions from microsomal reactions on major and minor metabolic pathways despite contributions from other processes such as conjugative reactions and transport on excretion of metabolites. The impact of 5F-APINACA modifications on targeting CB1 receptors lacks sufficient study in the field. To our knowledge, there are no studies on the affinity of hydroxy adamantyl metabolites for the receptors. Nevertheless, minor, oxidative defluorination and hydroxylation of the *N*-pentyl group lead to metabolites with lower CB1 affinity albeit similar to that for tetrahydrocannabinol (THC) while maintaining full agonist efficacy and possibly changing selective binding between CB1 and CB2 receptors [6]. These respective metabolic pathways are minor, so the overall impact on response to 5F-APINACA exposure is unclear. These metabolic pathways might still significantly contribute to toxicity of these abused drugs given that hydroxylation of a number of other structurally similar synthetic cannabinoids to 5F-APINACA leads to production of active metabolites. Most importantly, relevance of the outcomes would ultimately depend on factors altering relative contributions of competing oxidative pathways in 5F-APINACA metabolism.

Knowledge of enzymes responsible for competing metabolic pathways provides a basis for interpreting and predicting how clinical factors influence drug clearance and response. Our reported 5F-APINACA kinetics reflect an average adult liver but not the variabilities in metabolic capacities and corresponding responses in the general population due to differences in specific enzyme activities responsible for the observed reactions. Although not under steady-state conditions, prior screening studies by our group indicated major roles for CYP3A4/5 in hydroxylation of the adamantyl group of 5F-APINACA and possible minor roles for CYP2D6 and 2C8 [8]. We did not expand the study to phenotype P450s responsible for oxidation of the N-pentyl group; however, we can speculate on contributions from CYP2C and possibly CYP3A and 1A2 based on studies with the nonfluorinated APINACA and other similar synthetic cannabinoids as reviewed elsewhere [21]. P450 activities are influenced by clinical factors such as age, sex, genetic polymorphisms, and drug–drug interactions that would increase or suppress metabolism of 5F-APINACA and hence, response among abusers. Patterns correlating metabolism to abuse outcomes would likely reflect the dominance of CYP3A4/5 based on our reported kinetics for the efficiency of adamantyl group hydroxylation over oxidation of the N-pentyl moiety.

## 4. Materials and Methods

### 4.1. Materials

All chemical solvents, salts and buffers were purchased from Thermo Fisher Scientific (Waltham, MA, USA). NADPH-regenerating system components NADP disodium salt, glucose-6-phosphate dehydrogenase, and glucose-6-phosphate were purchased from Millipore-Sigma (St Louis, MO, USA), while magnesium chloride salt was purchased from Thermo Fisher Scientific. The substrate 5F-APINACA was provided by Dr. William Fantegrossi. Human liver microsomes pooled from 150 individuals were purchased from Corning (Corning, NY, USA). Dansylamide, used as the internal standard, was purchased from Millipore-Aldrich (St. Louis, MO, USA). ACD/ChemSketch 2017.2.1 software (Toronto, ON, Canada) was used for rendering structures of molecules.

### 4.2. Steady-State Kinetics for 5F-APINACA Metabolism by Human Liver Microsomes

The *in vitro* 5F-APINACA studies relied on reactions with human liver microsomes 150 (HLM150) as a model for the average adult human liver. The initiation of typical microsomal reactions involves addition of NAPDH or an NADPH-regenerating system; however, we observed significant oxidative defluorination of 5F-APINACA in the absence of NADPH for this drug [10] and other fluorinated synthetic cannabinoids [22,23,24]. Consequently, substrate 5F-APINACA was added last to the reaction. In a 96 half well plate, reaction conditions included human liver microsomes in 50 mM potassium phosphate buffer pH 7.4, 0.1% methanol (co-solvent), and an NADPH-regenerating system (0.4 U/mL glucose-6-phosphate dehydrogenase, 10 mM glucose 6-phosphate, 2 mM MgCl_2_, 500 μM NADP^+^) and was preincubated for 5 min at 37 °C with shaking at 350 rpm using a BMG Labtech THERMOstar incubator (Ortenberg, Germany). The addition of 5F-APINACA was used to initiate the reaction. For steady-state reactions, substrate concentrations were varied from 0 to 260 µM, selecting specific concentrations that best described its relationship with rate of turnover. Reactions were quenched by adding an equal volume of ice-cold methanol containing an internal standard (5 μM dansylamide final). Samples were chilled on ice for 10 min to optimize precipitation of proteins in phosphate buffer [25]. After 2800× *g* centrifugation at 4 °C for 15 min using a Sorvall ST 16R Centrifuge (Thermo Scientific, Waltham, MA, USA), the supernatant was transferred to a 96 well full-volume microplate for HPLC analyses.

For kinetic studies, initial control experiments were carried out to determine the linear response range for all observed metabolites as a function of protein concentration and time reflecting steady-state conditions. Based on these experiments, the optimal sensitivity under steady-state conditions corresponded to a reaction time of 30 min and protein concentration of 0.25 mg/mL HLM150 (data not shown). Each set of steady-state reactions were performed at least in triplicate and replicated two times. The composition of reactions was determined by liquid chromatographic analyses involving detection of analytes via UV/visible absorbance, fluorescence and mass spectroscopy (next section). Metabolite levels were then used to calculate initial reaction rates for plotting against substrate concentration with GraphPad Prism 8.0 from GraphPad Software, Inc (San Diego, CA, USA). The corresponding kinetic profiles were fit to multiple kinetic models (Michaelis–Menten, Michaelis–Menten plus linear phase, two combined Michaelis–Menten and Hill cooperativity) and the best-fit kinetic model and corresponding constants were determined using the extra sum-of-squares F test. In addition, we excluded statistically preferred mechanisms when best-fit values possessed open confidence intervals.

### 4.3. HPLC Resolution and Analysis of 5F-APINACA Analytes from Reactions

Sample reactions were analyzed to quantitate metabolites by fluorescence and then characterize their structures by mass spectrometry. As a first step, we designed an HPLC method to resolve the complex mixture of 5F-APINACA and its metabolites and enable their individual quantification. Analytes were separated on a Waters XBridge BEH C18 3.5 μM column (4.6 mm × 100 mm) using two LC instruments due to differences in attached detectors. A Shimadzu UHFLC was equipped with an SPD-10A UV–Vis (280 nm) and RF-10AXL fluorescence (excitation 280 nm, emission 650 nm) detectors. Alternatively, we used a Waters Acquity UPLC equipped with a 2475 FLR fluorescence (excitation 280 nm, emission 650 nm) and QDa single quadrupole detectors. QDa cone voltage was 20 V and the detector was set to detect a range from 150–650 *m*/*z* in the positive ion mode. The mobile phase consisted of solvent A (10% solvent B, 90% water) and solvent B (0.1% formic acid/acetonitrile). A gradient method started at 75% solvent A for the first minute, then decreased to 35% for the next 14 min. The method held 35% A for 3 min before returning to 75% over 3 min and holding for the final 4 min. The flow rate was 1.2 mL/min for a total run time of 25 min. Parent drug and metabolite responses were normalized to the internal standard dansyl amide and quantitated using a standard curve generated with 5F-APINACA assuming absence of any modification of the indazole ring to impact relative response. This assumption was validated by subsequent MS analysis of metabolites (vide supra).

The identity of the metabolites relied on two levels of mass spectral analyses. First, coupling of detectors with the Waters Acquity system provided a way to match fluorescent chromatographic peaks to parent masses for the analytes using the QDA single quadrupole detector. Second, a more detailed characterization of structure involved further MS analyses. For these assessments, sample supernatants after the previously described centrifugation step were transferred to a 96 well full-volume microplate and evaporated to dryness using an Organomation Microvap Nitrogen Evaporator System (Organomation Associates, Inc., Berlin, MA, USA). Dried wells were then resuspended in the mobile phase and the prepared samples were injected onto an Agilent Technologic 1290 Infinity HPLC using the same chromatographic method and column as described previously. Analytes were scanned with the Agilent Technologic 6490 Triple Quad LC–MS. The ESI source was operated in the negative and positive ion mode, and ion spectra were acquired in the full scan mode, monitoring the *m*/*z* range of 100–1200 amu. Subsequently, product ion spectra were generated from precursor ions with multiple reaction monitoring for fragmentation by collision-induced dissociation (20 eV) with a range of 45–1000 amu in the positive ion mode.

## 5. Conclusions

For the first time, we report the kinetics determining the metabolic flux of 5F-APINACA that alter its structure and, in doing so, determine potency and elimination for potential abusers. Based on observed regiospecificity of reactions, information on factors impacting hydroxylation of the adamantyl group and resulting effects on drug potency for CB1 interrogation are essential for understanding the effects on abuse response. Unfortunately, those types of studies have primarily focused on N-pentyl modifications that may not contribute significantly to outcomes due to its less significant occurrence in metabolism. More research is clearly needed in this area. This critical guiding insight was made possible from the novel application of an analytical approach to infer metabolite levels for kinetic studies. We leveraged the unmetabolized central indazole core of 5F-APINACA for quantitation by absorbance rather than the commonly used MS response. In fact, we showed how MS responses varied among metabolites. Consequently, its use in “semi-quantitative” inference studies over- or under-predicts actual metabolites levels, leading to inaccuracies in the data. Importantly, the indazole chromophore for 5F-APINACA is common among synthetic cannabinoids. Our novel application could then be applied universally to generate more accurate assessments of synthetic cannabinoid metabolism, and thus develop more appropriate models to interpret and predict abuser outcomes from these illicit drugs.

## Figures and Tables

**Table 1:** Analyte MS data from 5F-APINACA metabolism by human liver microsomes.

ID	t_R_^a^	QDa ^b^ Precursor [M + H]^+^ (*m*/*z*)	QQQ ^c^ Precursor [M + H]^+^ (*m*/*z*)	Product Ions (*m*/*z*)	Proposed Modifications	Metabolite Order
**M1**	2.17	ND	414.4	131.0, 149.0, 167.0, 230.0, 248.0	Dihydroxy adamantyl Oxidative defluorination	Tertiary
**M2**	2.24	ND	432.4	248.9, 167.1, 149.0	Dihydroxy adamantyl Other hydroxylation	Tertiary
**M3**	3.76	398.26	398.4	151.1, 133.0	Monohydroxy adamantyl Oxidative defluorination	Secondary
**M4**	5.66	416.26	416.3	233.1, 167.0, 149.1, 131.0	Dihydroxy adamantyl	Secondary
**M5**	8.32	400.29 ^d^	400.4	151.1, 133.0	Monohydroxy adamantyl	Primary
**M6**	8.42	400.29 ^d^	400.2	151.0, 133.0	Monohydroxy adamantyl	Primary
**M7**	9.10	400.29 ^d^	400.2	151.0, 133.0	Monohydroxy adamantyl	Primary
**M8**	11.9	404.28	382.2	135.0	Oxidative defluorination	Primary
**M0**	17.9	384.28	384.2	135.0	None	Parent

^a^ Retention time (min) corresponding to analysis by an Agilent Technologic 1290 Infinity HPLC equipped with a 6490 Triple Quad MS. ^b^ Data obtained using a Waters Acquity UPLC-Fluorescence/QDa (single quadrupole). ND, not detected. ^c^ Data obtained using an Agilent Technologic 1290 Infinity HPLC equipped with a 6490 Triple Quad MS. ^d^ Waters Acquity UPLC-Fluorescence/QDa instrument only detected one of three isomers for the hydroxy adamantyl metabolites, when compared to the more sensitive Agilent Technologic 1290 Infinity HPLC equipped with a 6490 Triple Quad MS instrument.

**Table 2:** Steady-state kinetics for 5F-APINACA metabolism by human liver microsomes ^a^.

Peak ID	Mechanism	Kinetic Constants				
		V_max_	K_m_ (μM)	K_i_ (μM)	V_max2_	K_m2_ (μM)
**M3**	Substrate inhibition	16	4.3	92	-	-
		(12–19)	(1.8–6.8)	(39–145)		
**M4**	Substrate inhibition	106	1.6	480	-	-
		(98–114)	(1.1–2.1)	(270–700)		
**M5**	Michaelis–Menten + linear	11	4.4	-	0.021	-
		(9.0–12)	(2.7–6.9)		(0.012–0.030)	
**M6/M7 ^b^**	Michaelis–Menten + linear	230	9.7	-	0.18	-
		(227–234)	(9.1–10)		(0.16–0.20)	
**M5 + M6/M7 ^c^**	Michaelis–Menten + linear	200	4.6	-	0.41	-
		(170–230)	(2.8–7.4)		(0.20–0.60)	
**M4 + M5 + M6/M7 ^d^**	Michaelis–Menten + linear	280	3.1	-	0.38	-
		(250–320)	(2.0–4.6)		(0.16–0.60)	
**M8**	Biphasic	22	4.2	-	60.1	149
		(21–23)	(3.9–4.6)		(59.7–60.6)	(143–157)

^a^ For each metabolite, the reported mechanism reflects the most statistically preferred model. Confidence intervals for corresponding constant are shown in parentheses. V_max_ units are pmol/min/mg protein. ^b^ Individual metabolites detected with an Agilent Technologic 1290 Infinity HPLC equipped with a 6490 Triple Quad MS, but were not detected using the Waters instrument. ^c^ Kinetics for hydroxy adamantyl isomers (M5 and M6/M7) combined for global analysis of that initial step in 5F-APINACA metabolism. ^d^ Kinetics for total adamantyl group oxidation including mono- and dihydroxylated metabolites.
